# Plasma ctDNA enhances the tissue-based detection of oncodriver mutations in colorectal cancer

**DOI:** 10.1007/s12094-024-03422-7

**Published:** 2024-05-22

**Authors:** Wei Wang, Yisen Huang, Jianqiao Kong, Lin Lu, Qianxiu Liao, Jingtao Zhu, Tinghao Wang, Linghua Yan, Min Dai, Zhan Chen, Jun You

**Affiliations:** 1 The First People’s Hospital of Foshan, Foshan, 528000 Guangdong China; 2 Department of Gastroenterology, Quanzhou First Hospital Affiliated to Fujian Medical University, Quanzhou, 362002 Fujian China; 3 Department of General Surgery, Xiangyang No.1 People’s Hospital, Hubei University of Medicine, Xiangyang, 441000 Hubei China; 4 Colorectal Surgery Department, General Hospital of Ningxia Medical University, Yinchuan, 750001 Ningxia China; 5 Department of Laboratory Medicine, Chengdu First People’s Hospital, Chengdu, 610041 Sichuan China; 6 The Third Clinical Medical College, Fujian Medical University, Xiamen, 361001 Fujian China; 7 Shanghai Tongshu Biotechnology Co., Ltd, Shanghai, 201900 China; 8 Department of Pathology, Wuhu Hospital, East China Normal University (The Second People’s Hospital, Wuhu), Wuhu, 241000 Anhui China; 9 Department of General Surgery, Chenggong Hospital of Xiamen University School of Medicine, Xiamen, 361001 Fujian China; 10 Department of Gastrointestinal Oncology Surgery, Cancer Center, The First Affiliated Hospital of Xiamen University, School of Medicine, Xiamen University, Xiamen, 361001 Fujian China

**Keywords:** Colorectal cancer, Circulating tumor DNA, Tumor mutational burden, Concordance, Positive mutation rate

## Abstract

**Purpose:**

The advent of circulating tumor DNA (ctDNA) technology has provided a convenient and noninvasive means to continuously monitor cancer genomic data, facilitating personalized cancer treatment. This study aimed to evaluate the supplementary benefits of plasma ctDNA alongside traditional tissue-based next-generation sequencing (NGS) in identifying targetable mutations and tumor mutational burden (TMB) in colorectal cancers (CRC).

**Methods:**

Our study involved 76 CRC patients, collecting both tissue and plasma samples for NGS. We assessed the concordance of gene mutational status between ctDNA and tissue, focusing on actionable genes such as *KRAS*, *NRAS*, *PIK3CA*, *BRAF*, and *ERBB2*. Logistic regression analysis was used to explore variables associated with discordance and positive mutation rates.

**Results:**

In total, 26 cancer-related genes were identified. The most common variants in tumor tissues and plasma samples were in *APC* (57.9% vs 19.7%), *TP53* (55.3% vs 22.4%) and *KRAS* (47.4% vs 43.4%). Tissue and ctDNA showed an overall concordance of 73.53% in detecting actionable gene mutations. Notably, plasma ctDNA improved detection for certain genes and gene pools. Variables significantly associated with discordance included gender and peritoneal metastases. TMB analysis revealed a higher detection rate in tissues compared to plasma, but combining both increased detection.

**Conclusions:**

Our study highlights the importance of analyzing both tissue and plasma for detecting actionable mutations in CRC, with plasma ctDNA offering added value. Discordance is associated with gender and peritoneal metastases, and TMB analysis can benefit from a combination of tissue and plasma data. This approach provides valuable insights for personalized CRC treatment.

**Supplementary Information:**

The online version contains supplementary material available at 10.1007/s12094-024-03422-7.

## 1 Introduction

Colorectal cancer (CRC) stands as the third most commonly diagnosed cancer and the second leading cause of cancer-related deaths in 2020, accounting for approximately 9.8% (over 1,880,000) of new cancer cases and 9.2% (over 915,000) of cancer fatalities in 2020 [1]. The initiation and progression of CRC are widely attributed to the presence of gene mutations in several oncogenes, prominently including *KRAS*, *NRAS*, *BRAF*, *PIK3CA* and *HER2 (ERBB2)*, etc. [2–9]. The accurate and dynamic identification of these therapeutically targetable mutations holds significant promise for precision-based personalized treatment in CRC, ultimately enhancing patient outcomes and prognosis.

Tumor tissue next-generation sequencing (NGS) of clinically targetable mutations is important for precise treatment for CRC, while intertumor and intratumor heterogeneity hampers the further application of tissue-based NGS [5, 10]. In addition, tumor tissues are not always available or eligible for NGS. Recently, developed circulating tumor DNA (ctDNA) technique as a convenient and noninvasive means has been rapidly employed for dynamically obtaining and monitoring landscape of genomic information to instruct personalized cancer treatment [11, 12], which has a concordant detection efficacy with the matched tumor tissue NGS [13] but overcomes the influence of intratumor heterogeneity [14] affecting tissue NGS. ctDNA has been effectively applying in the identification of clinically relevant mutations. For example, Hsu et al. targeted ctDNA to monitor genetic variants and response to therapies and predict prognosis in CRC [15, 16]. Tarazona et al. detected plasma post-surgery ctDNA to track minimal residual disease and identify a high risk of relapse in patients with localized colon cancer, which showed post-surgery ctDNA detection was correlated with poor disease- free survival and that presence of ctDNA post therapy in patients receiving adjuvant chemotherapy was associated with early relapse [17]. Xu et al. explored the application of ctDNA in the assessment of clinical tumor mutation burden (TMB) in Chinese patients with metastatic CRC [18].

Not only does ctDNA detect the same mutations as tissue (accounting for most of those detected), but it can also distantly identify some mutations that might be omitted by the other method. For example, Takeda et al. showed that in 34 untreated CRC patients, 53 mutations were detected in tumor tissues, and 47 mutations were detected in ctDNA, 20 of which were undetected in tissues [19]. Cao et al. reported among 59 mutations in 11 advanced CRC tissues, 52 (88.14%) was also identified in matched blood, while 19 mutations in plasma ctDNA were missed in the corresponding tissues [20]. It appears that plasma ctDNA could detect more cancer mutations in CRC than tissues. In addition, detection concordance has been observed between some important clinically relevant gene mutations in tissues and ctDNA. For example, some reports showed a high concordance (77%) of *KRAS* variant between tumor tissues and plasma [21], and concordance between tissue and blood ctDNA ranged from 63.2% (*APC*) to 85.5% (*BRAF*) in CRC [22].

To date, there is a lack of consistency in studies regarding the positive detection of clinically targetable mutations, both at the comprehensive and individual levels, with limited investigations into the application of plasma ctDNA for evaluating TMB status in CRC. In this study, we systematically assess the detection efficacy of tissue versus plasma ctDNA, along with the additional value of plasma ctDNA in comparison to tissue-based NGS, for therapeutically targetable mutations (at both comprehensive and individual levels) and TMB-H in Chinese CRC patients.

## Materials and methods

### Patients and sample collection

We conducted a retrospective review and enrolled 76 CRC patients who underwent surgical resection at eight hospitals. Peripheral blood samples were collected from these patients prior to treatment, and none had received radio chemotherapy before the sample collection or surgical tumor resection. Additionally, the patients had an adequate quantity and quality of tissue DNA for NGS analysis. Those individuals with concurrent cancer types were excluded from the study. The diagnosis of all the samples was performed by two experienced molecular pathologists based on the morphology of hematoxylin & eosin staining (HE), and the tumor cell content was higher than 50%. This study was approved by the ethics committees of the corresponding hospitals. Written informed consent was obtained from all enrolled patients.

### DNA extraction and sequencing

Tissue DNA was extracted from the FFPE tissues using the QIAamp DNA FFPE tissue kit (Qiagen). The plasma DNA was extracted using a DNeasy Blood & Tissue kit (Qiagen) according to the manufacturer’s instructions. The resultant DNA was then quality-controlled using Nanodrop and Qubit (Thermo Fisher Scientific) to ensure adequate purity and quality. Illumina paired-end libraries were prepared from extracted DNA and sequenced on Illumina HiSeq platforms. The 556 or 105 panel produced by Shanghai Tongshu Biotechnology Co., Ltd. was used as a DNA capture probe of cancer-related genes. All the tumor tissues and plasma samples were subjected to NGS of driver mutated genes. The average sequencing depth in tissues is ≥ 1000 × and the average sequencing depth in plasma cfDNA is ≥ 7000 × . The variant allele frequency (VAF) is ≥ 1% for tissue DNA and ≥ 0.1% for cfDNA from plasma. BWA (Burrows-Wheeler-Alignment) software was used to compare the sequencing data. GATK (The Genome Analysis Toolkit), MuTect [23] and VarScan [24] were used to alignment optimization, variant calling and annotation, respectively.

The quantification of ctDNA levels followed a previously established method [25, 26]. This involved calculating the maximum variant allele frequency (maxVAF) and then determining the ctDNA concentration (in haploid genomic equivalents per milliliter, hGE/mL) using the formula: ctDNA concentration (hGE/mL) = (mean ctDNA VAF * cell-free DNA concentration (pg/mL))/3.3, assuming that each haploid genomic equivalent (hGE) weighed 3.3 pg.

### Statistical analysis

Detected mutations with allele abundance of ≥ 0.1% were recorded. Samples that were identified with at least one mutation in oncodrivers by any of the tissue and plasma assays were considered true positive, and those showing negative by both assays were considered true negative [27]. The concordance was defined as the number of concordant positive and negative cases/total cases × 100%, positive detection rate was calculated as the positively detected case number/total case number × 100%, and sensitivity was shown as the detected case number of true positive/total true positive case number × 100%. Additionally, the TMB analysis exclusively employed sequencing data from the panel of 556 cancer-related genes, utilizing the upper quartile TMB value of the tissue sample as the threshold to distinguish TMB levels. The software SPSS 25.0 (IBM Corp., Armonk, NY, USA) was used for all statistical analysis. χ^2^ test was used in the univariate analysis of ctDNA-positive rate and concordance detection. Statistical significance was considered when *P* < 0.05.

## Results

### Patient characteristics

Seventy-six CRC patients were successfully enrolled in the study, and tissue and plasma samples were collected and analyzed separately for NGS. Within our cohort, there were 49 males (64.47%) and 27 females (35.53%); ages ranged from 33 to 80 (median: 63). The majority of patients had stage III (48, 63.16%), and the remainder had stage II (11, 14.47%) or IV (17, 22.37%). Fifty-nine patients (77.63%) had no distant metastasis, and 17 patients (22.37%) had distant metastasis at different sites including liver, lung, peritoneum, navel, etc. In addition, 4 patients were DNA mismatch repair (MMR) deficient (dMMR), and 72 were MMR proficient (pMMR), where MMR status was identified by immunohistochemistry (IHC). The basic information of the patients was shown in Table 1.

**Table 1:** Baseline demographic and clinical characteristics (*N* = 76)

Characteristics		Number	Percentage (%)
Gender	Male	49	64.47
	Female	27	35.53
Age (median: 63 years)	≥ 60	43	56.58
	< 60	33	43.42
Stage at diagnosis	IIa/IIb/IIc	11	14.47
	IIIa/IIIb/IIIc	48	63.16
	IV	17	22.37
Primary site	Colon	68	89.47
	Rectum	6	7.89
	colorectal	2	2.63
The degree of differentiation	Moderately	64	84.21
	Poorly	12	15.79
Distant metastasis	No	59	77.63
	Yes	17	22.37
Metastatic site	Liver	11	14.47
	Lung	5	6.58
	Node	62	81.58
	Peritoneal	4	5.26
	Navel	1	1.32
Number of metastatic sites	0	9	11.84
	1	53	69.74
	≥ 2	14	18.42
Status of MMR	dMMR	4	5.26
	pMMR	72	94.74

### Concordance of gene mutational status between ctDNA and tissue

**Fig. 1:**
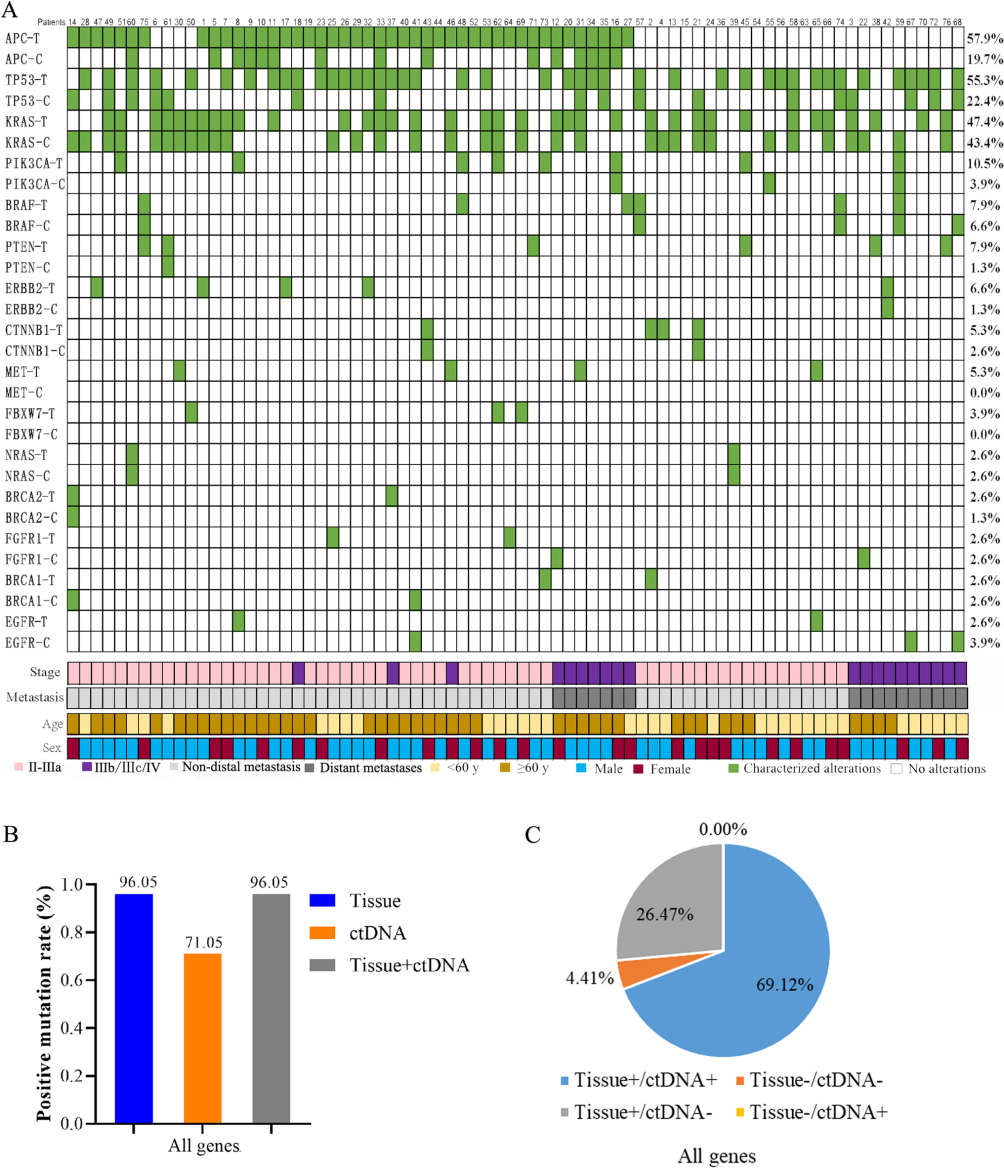
Concordance of mutation landscape between tissue and plasma ctDNA. **A** The mutational landscapes of tissue and plasma ctDNA are provided along with the most frequently mutated oncodrivers. **B** A comparison of the positive mutation rate of all genes between tissue and plasma ctDNA. **C** Concordance of all genes between tissue and plasma ctDNA

**Fig. 2:**
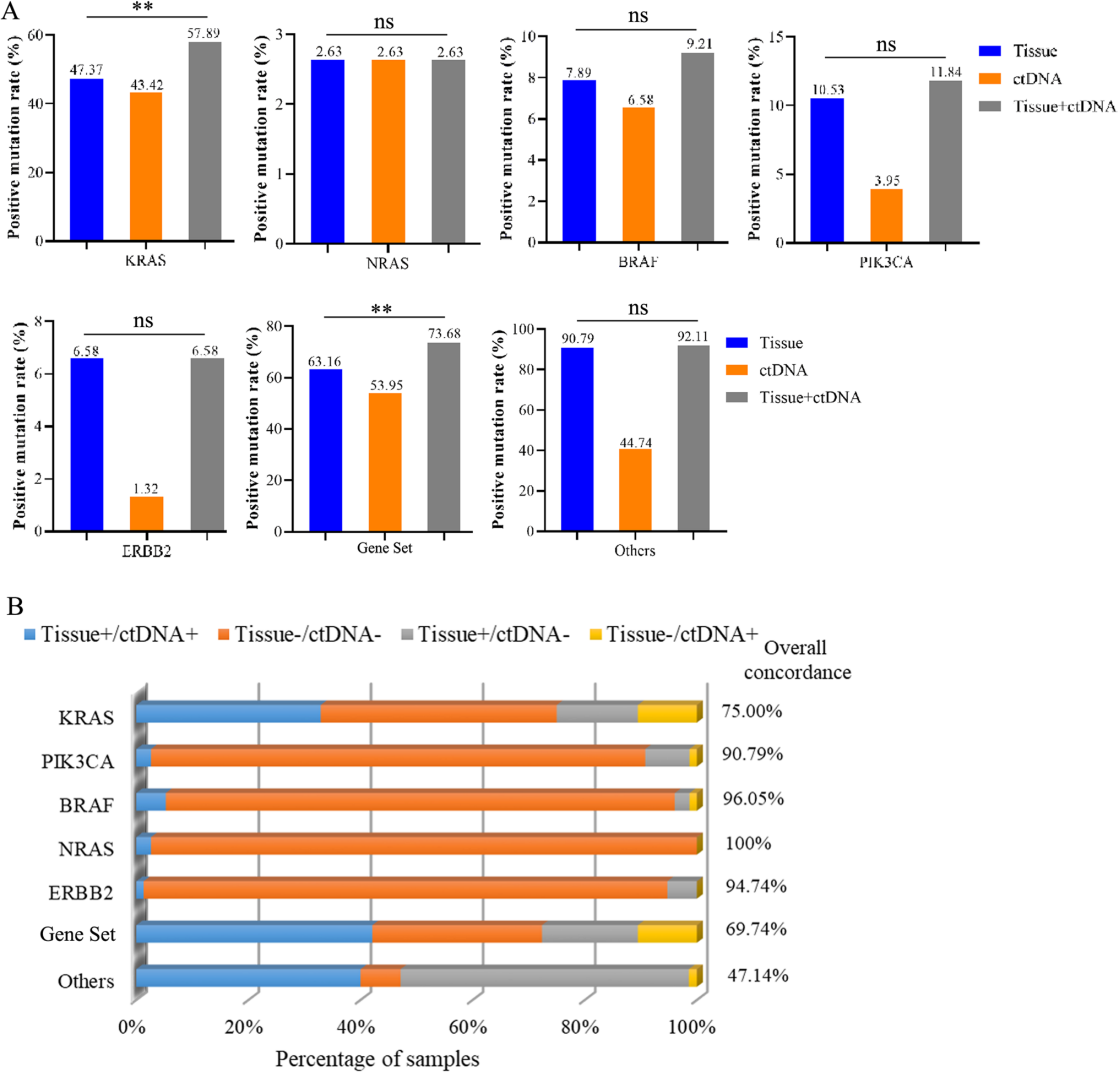
Concordance of KRAS/PIK3CA/BRAF/NRAS/ERBB2 between tissue and plasma ctDNA. **A** A comparison of the positive mutation rate of *KRAS*/*PIK3CA*/*BRAF/NRAS*/*ERBB2* between tissue and plasma ctDNA. **B** Concordance of *KRAS*/*PIK3CA*/*BRAF/NRAS*/*ERBB2* between tissue and plasma ctDNA. *p < 0.05; **p < 0.01; ns, no significant difference

In total, 26 cancer-related genes were found in tissue or plasma samples from these 76 CRC patients. As shown in Fig. 1a, the most frequent variants occurred in *APC* (57.9% vs 19.7%), *TP53* (55.3% vs 22.4%) and *KRAS* (47.4% vs 43.4%), both in tumor tissue and plasma ctDNA samples. The mutation type of each oncodriver was summarized in Supplementary Table 1. The positive detection rates of tissue and ctDNA were 96.05% and 71.05%, respectively, with an overall consistency of 73.53% (Fig. 1B, C). Here, we focused on the genes *KRAS*, *NRAS*, *PIK3CA*, *BRAF* and *ERBB2*, where mutations detected were considered potentially actionable. The positive mutation rate of plasma plus tissue testing for these genes was not lower than that of a single assay, either for individual genes or gene pools as shown in Fig. 2A. Particularly, plasma ctDNA significantly enhances the tissue-based detection of *KRAS* and *KRAS/NRAS/BRAF/PIK3CA/ERBB2* (McNemar’s test, p < 0.01) (Supplementary Table 2). The overall concordance of *KRAS*, *NRAS*, *PIK3CA*, *BRAF* and *ERBB2* between plasma- and tissue-based analyses was 75% (57/76), 90.79% (69/76), 96.05% (73/76), 100% (76/76) and 94.74% (72/76) (Fig. 2b). The concordance analysis of all detected genes is presented in Supplementary Table 3.

### Variables associated with discordance and positive mutation rate

**Fig. 3:**
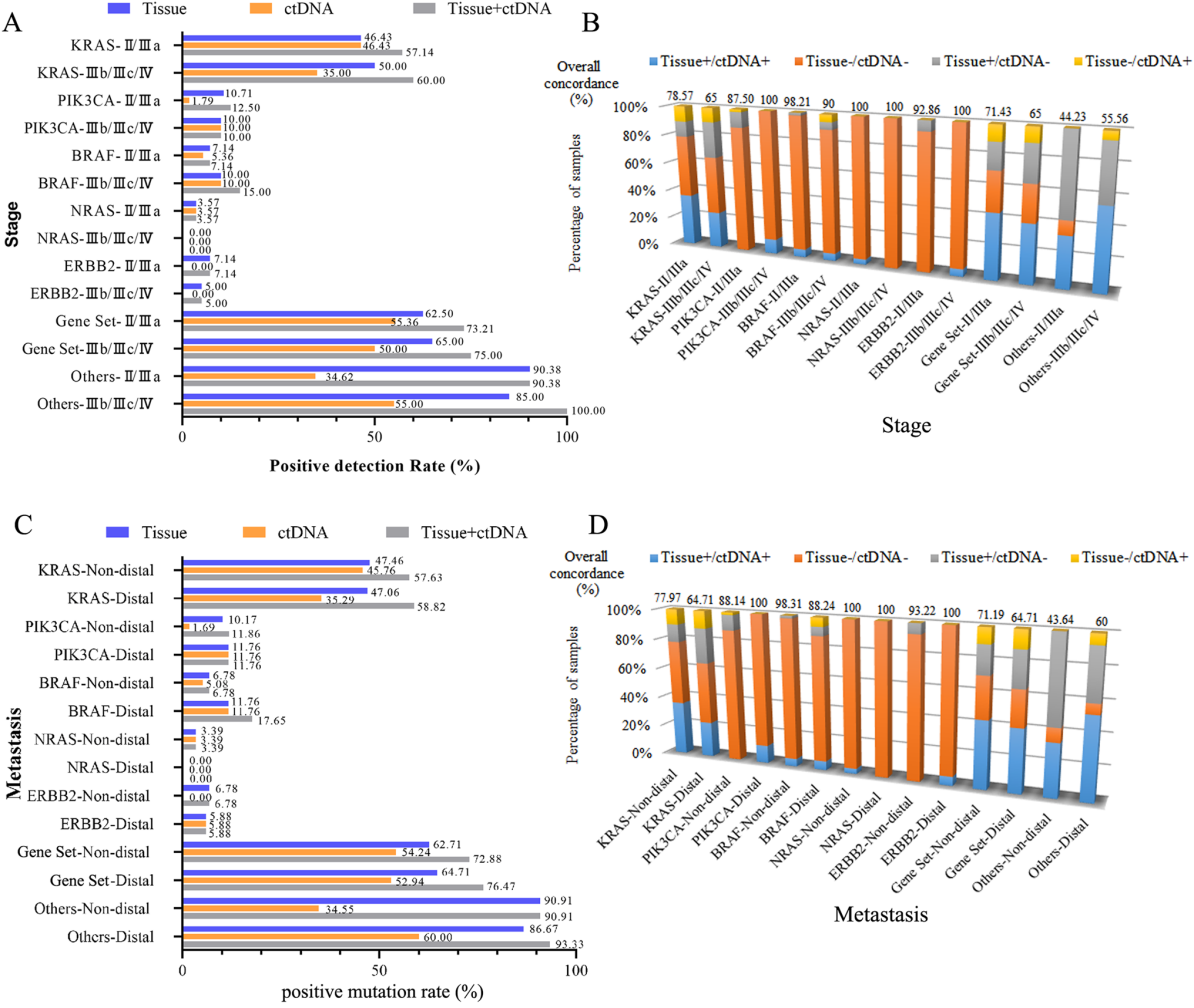
Variables associated with discordance and positive mutation rate of KRAS/PIK3CA/BRAF/NRAS/ERBB2 between tissue and plasma ctDNA. **A** The positive mutation rate of *KRAS*/*PIK3CA*/*BRAF/NRAS*/*ERBB2* between tissue and plasma ctDNA from patients with different stages. **B** Concordance of *KRAS*/*PIK3CA*/*BRAF/NRAS*/*ERBB2* between tissue and plasma ctDNA from patients with different stages. **C** The positive mutation rate of *KRAS*/*PIK3CA*/*BRAF/NRAS*/*ERBB2* between tissue and plasma ctDNA from patients with distal metastasis or not. **D** Concordance of *KRAS*/*PIK3CA*/*BRAF/NRAS*/*ERBB2* between tissue and plasma ctDNA from patients with distal metastasis or not

In addition, variables associated with the discordance and positive mutation rate of tissue and ctDNA were analyzed. The overall positive mutation rate of *KRAS*/*PIK3CA*/*BRAF/NRAS*/*ERBB2* for advanced patients was higher than early patients, and those with distal metastasis were higher than those without distal metastasis, but there was no statistically significant difference (Fig. 3). The increased positive mutation rate of combined tissue and plasma testing was independent of the clinical characteristics of the patients (Table 2). We investigated the logistic regression analysis for identifying the variables associated with the discordance. Stage and metastatic status did not appear to be significantly associated with inconsistency (Fig. 3 and Table 3). The discordance showed a strong association with gender (*P* = 0.030) and peritoneal metastases (*P* = 0.045).

**Table 2:** Variable analysis of the positive mutation rate of KRAS/NRAS/ PIK3CA/BRAF/ERBB2 using ctDNA and tissue DNA (*N* = 76)

Variables	Positive mutation rate					P value
	Tissue		ctDNA		Combination	
Age						0.719
< 60	57.58% (19/33)		60.61% (20/33)		75.76% (25/33)	
≥ 60	67.44% (29/43)		48.84% (21/43)		72.09% (31/43)	
Gender						0.954
Male	63.27% (31/49)		44.90% (22/49)		73.47% (36/49)	
Female	62.96% (17/27)		70.37% (19/27)		74.07% (20/27)	
Stage						0.876
II/IIIa	62.5% (35/56)		55.36% (31/56)		73.21% (41/56)	
IIIb/IIIc/IV	65% (13/20)		50% (10/20)		75% (15/20)	
Primary site						0.687
Colon	61.76% (42/68)		52.94% (36/68)		72.06% (49/68)	
Rectum	83.33% (5/6)		66.67% (4/6)		100% (6/6)	
Colorectal	50% (1/2)		50% (1/2)		50% (1/2)	
The degree of differentiation						0.910
Moderately	62.50% (40/64)		53.13% (34/64)		73.44% (47/64)	
Poorly	66.67% (8/12)		58.33% (7/12)		75.00% (9/12)	
Distant metastasis						0.767
No	62.71% (37/59)		54.24% (32/59)		72.88% (43/59)	
Yes	64.71% (11/17)		52.94% (9/17)		76.47% (13/17)	
Number of metastatic sites						0.422
0	77.78% (7/9)		88.89% (8/9)		88.89% (8/9)	
1	58.49% (31/53)		49.06% (26/53)		67.92% (36/53)	
≥ 2	71.43% (10/14)		50.00% (7/14)		78.57% (11/14)

**Table 3:** Variable analysis of the consistency between ctDNA and tissue DNA detection for KRAS/NRAS/PIK3CA/BRAF/ERBB2 (*N* = 76)

Variables	Inconsistent (*n* = 23)	Consistent (*n* = 53)	χ2 value	*P* value
Age			0.261	0.610
< 60	11	22		
≥ 60	12	31		
Gender			4.736	**0.030**
Male	19	30		
Female	4	23		
Stage			0.289	0.591
II/IIIa	16	40		
IIIb/IIIc/IV	7	13		
Primary site			0.000	0.987
Colon	20	48		
Rectum	3	3		
Colorectal	0	2		
The degree of differentiation			0.187	0.665
Moderately	20	44		
Poorly	3	9		
Distant metastasis			0.263	0.608
No	17	42		
Yes	6	11		
Metastatic site				
Liver metastasis			4.430	0.098
No	22	43		
Yes	1	10		
Lung metastasis			2.243	0.134
No	20	51		
Yes	3	2		
Peritoneal metastasis			4.004	**0.045**
No	20	52		
Yes	3	1		
Number of metastatic sites			0.050	0.824
0	3	6		
1	15	38		
≥ 2	5	9		

Bold font indicates statistical significance, *P* < 0.05

### Concordance of TMB-H between ctDNA and tissue

**Fig. 4:**
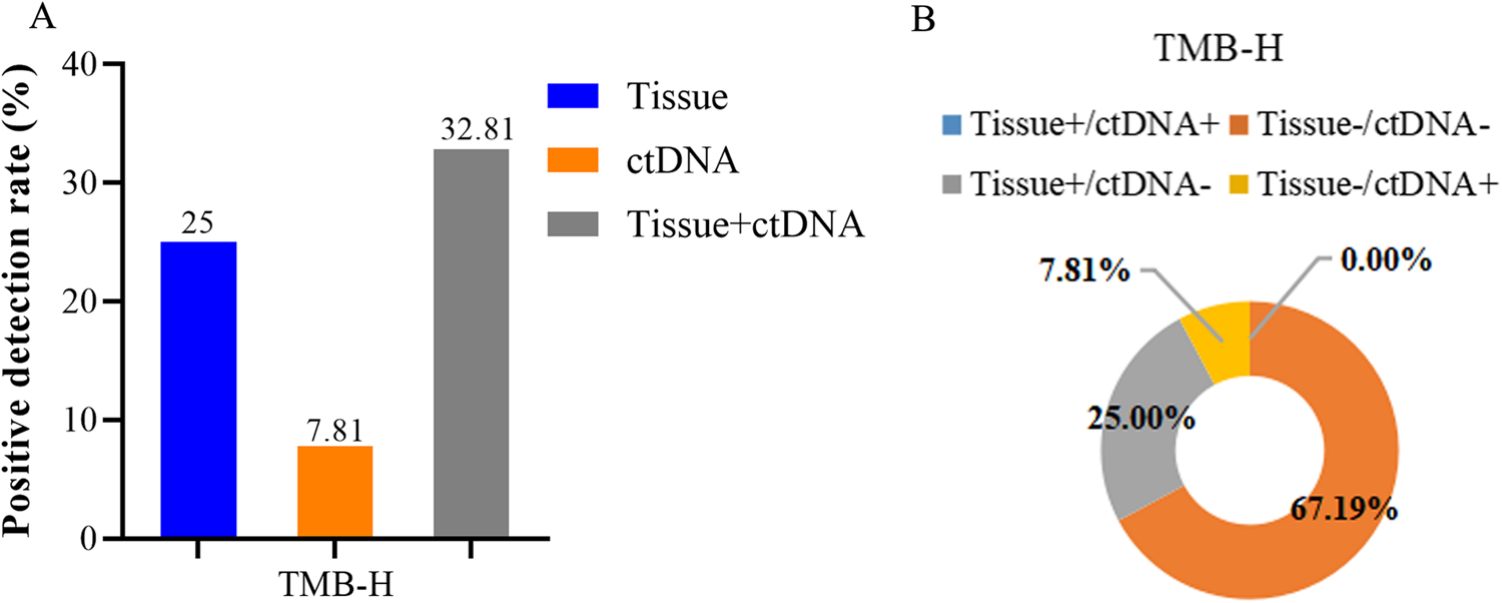
Concordance of TMB-H between tissue and plasma ctDNA. **A** Comparison of the positive detection rate of TMB-H between tissue and plasma ctDNA. **B** Concordance of TMB status between tissue and plasma ctDNA

TMB was then classified into TMB-H and TMB-L according to the upper quartile TMB of 9.48 mutations/Mb (TMB ≥ 9.48 was defined as TMB-H and those < 9.48 as TMB-L). For the detection of TMB-H, the positive detection rates of TMB-H by tissues and plasma were 25% (16/64) and 7.81% (5/64), respectively, and plasma plus tissue increased the detection rate to 32.81% ([16 + 5]/64), and the overall concordance was 67.19% (43/64) (Fig. 4 and Supplementary Table 4).

## 4 Discussion

**Fig. 5:**
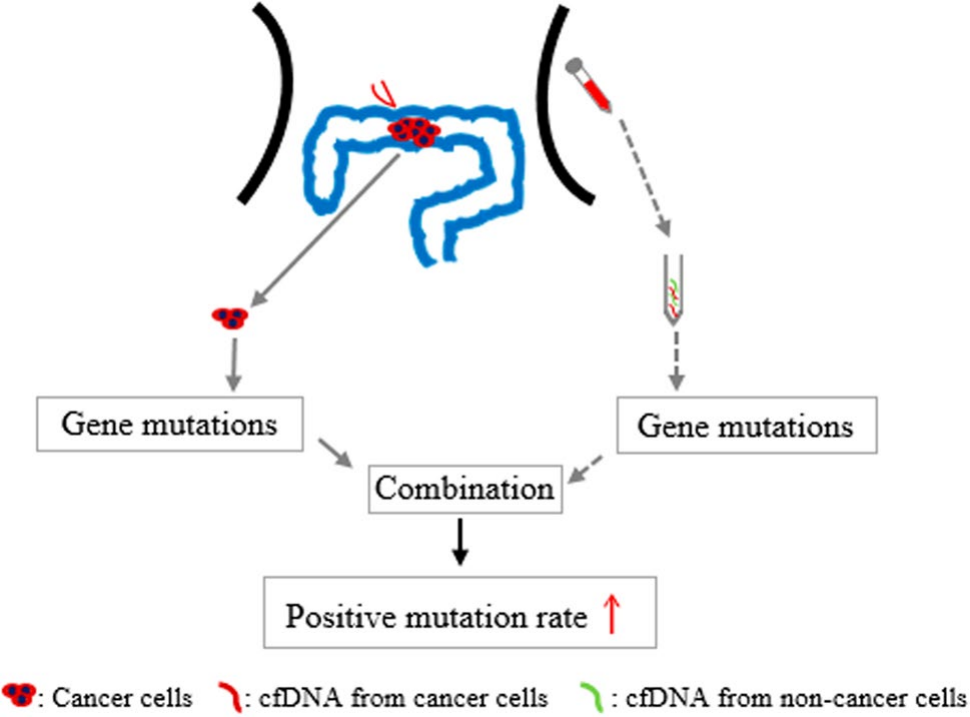
Schematic diagram of the study

Currently, studies regarding the positive detection of clinically targetable mutations in CRC by ctDNA, at both the whole and individual mutation levels, are not consistent, and there were few studies reporting the application of plasma ctDNA in monitoring TMB in CRC. In this study, we aimed to assess the concordance between gene mutational status in ctDNA and tissue samples from CRC patients and to determine the impact of combining these two modalities in detecting actionable mutations. Our findings shed light on the utility of plasma ctDNA as a complementary tool for assessing genetic alterations in CRC patients. We also evaluated the concordance of TMB-H classification between ctDNA and tissue samples. The progression of driver gene alterations in CRC represents a stepwise tumorigenesis process. It’s noteworthy that less than 1% of human genes are likely to undergo transformation into cancer-driver genes, which play an active role in regulating cell survival and fate, consequently impacting the stability of normal genomes [28, 29]. In contrast to diseases like cystic fibrosis or muscular dystrophy, where cancer does not stem from a single gene defect, it’s more accurate to regard altered cancer genes as contributory factors rather than root causes of cancer. Nevertheless, studies have revealed that several frequently mutated genes in CRC, such as *APC*, *TP53*, *KRAS*, and *BRAF*, are not only significantly influenced by individual somatic mutations but also exert a substantial functional impact [30]. In our investigation, we identified a total of 26 cancer-related genes, with *APC*, *TP53*, and *KRAS* exhibiting the most prevalent mutations in both tumor tissue and plasma samples. The *APC* gene is recognized as the sentinel gene in CRC [31]. *KRAS* plays a pivotal role in promoting cancer through the activation of *RAF*-*MAPK* and *PI3K* pathways. Typically, *APC* mutations coincide with *KRAS* or *TP53* mutations, or both, as corroborated by our findings. The overall concordance in detecting mutations in these tumor-related genes stood at 73.53%, signifying robust consistency between tissue- and plasma-based NGS. This level of concordance surpasses that reported in some earlier studies [18, 32]. Additionally, we identified other potentially actionable target genes, including *NRAS*, *PIK3CA*, *BRAF*, and *ERBB2*, which are closely linked to anti-*EGFR* resistance. The concordance between individual gene mutations from tissue- and plasma-based NGS exceeded 90%, with *NRAS* achieving a perfect 100% agreement, slightly exceeding results in certain prior studies [33–35]. Furthermore, even though the positive mutation rates of *KRAS*, *BRAF*, *PIK3CA*, and *ERBB2* were higher in tissue samples, the combination of plasma and tissue data resulted in a higher overall positive detection rate. This underscores that both tissue and plasma ctDNA NGS are effective in identifying therapeutically targetable mutations in CRC. Notably, the increased positive mutation rate achieved through the combined tissue and plasma testing was independent of the clinical characteristics of the patients. In summary, plasma ctDNA adds significant value to routine tissue NGS, as illustrated in Fig. 5.

We conducted a comprehensive analysis of variables influencing concordance. Discordance could potentially arise from the low levels of ctDNA shedding by tumors. Our investigation revealed a higher positive mutation rate of *KRAS*/*PIK3CA*/*BRAF*/*NRAS*/*ERBB2* in advanced-stage patients compared to early-stage patients. Likewise, patients with distal metastasis exhibited a higher positive mutation rate than those without distal metastasis, although this difference did not reach statistical significance. This observation suggests that later disease stages may yield more ctDNA release than earlier stages. Notably, the logistic regression analysis revealed that peritoneal metastases were a significant variable associated with discordance, while liver and lung metastases showed no significant association with discordance in our cohort. Hideaki Bando et al. found that lung metastasis alone was the most significant factor associated with discordance [34]. Due to our limited sample size, the difference in discordance between patients with lung metastasis and patients with peritoneal metastasis was only one person. Therefore, these findings should be further substantiated with a larger cohort in future studies. Additionally, the plausible reason for discordance could be lower ctDNA shedding from different tumors [34]. Some studies aslo reported that in recurrence CRCs, ctDNA detection was challenging for lung metastases and peritoneal metastases [34, 36]. This discordance between tissue- and plasma-based NGS is not the factors mentioned above; it’s also influenced by the patients’ treatment history or the differences in tumor heterogeneity, even the bias in multicenter patients [34–36].

To account for spatial and temporal variability, it is imperative to periodically assess the genomic profile of CRC patients throughout their treatment. Liquid biopsies can play a pivotal role in profiling the patient’s specific molecular makeup, particularly when considering anti-*EGFR* treatment options [37, 38]. One of the strongest arguments favoring this minimally invasive approach is the ability to perform *RAS*/*RAF* testing at the point of decision-making. Within our cohort, we observed instances where 11 and 2 patients had tissue mutations in *KRAS* and *BRAF* that were undetectable in plasma, and conversely, eight and one patients had plasma mutations in *KRAS* and *BRAF* that were absent in tissue samples. The absence of *RAS*/*RAF* mutations in plasma might be attributed to biological factors influencing ctDNA release—a crucial area for further investigation. This issue becomes even more pronounced in light of false-negative results, which pose a significant challenge in plasma testing for *RAS*/*RAF* mutations. Negative interactions may occur between anti-*EGFR* drugs and oxaliplatin-based regimens in patients with *RAS*/*RAF* mutations. Similar trends were observed in the detection of other genes, including *PIK3CA* and *ERBB2* mutations, within our cohort. Additionally, the detection rate of TMB-H in tissue samples was 25%, significantly lower at 7.81% in plasma, with poor consistency. We suspect several factors may contribute to this result. In our study, a significant proportion of patients (59 cases, 77.6%) were classified as stage II/III. The observed low TMB values in ctDNA may be attributed to restrictions on the release of tumor-derived DNA into the bloodstream and factors related to the early stages of the disease. Consequently, the TMB levels in ctDNA (with 92.2% of patients in this study having TMB < 10 mutations/Mb) are significantly lower than tissue TMB levels. In the absence of uniform TMB grading criteria, it is necessary to determine the cutoff value for defining TMB as "TMB-high," which may also be one of the reasons for the bias. Furthermore, compared to TMB estimates based on tumor tissue, TMB estimates based on ctDNA cannot effectively avoid interference from lineage mutations; thus, it may lead to calculation biases, especially in patients with relatively low mutation counts. Despite this, the combined analysis of plasma and tissue increased the positive detection rate to 32.81%. This outcome underscores that for detecting TMB-H in CRC, plasma ctDNA alone may not suffice; nevertheless, it effectively enhances the positive detection rate when used in conjunction with tissue samples. Studies have established a correlation between TMB and ctDNA levels. Lower TMB values are associated with a higher likelihood of false-negative plasma ctDNA results and reduced concordance between tissue and plasma ctDNA detection [39].

## Conclusion

Our study highlights the potential clinical implications of combining tissue and plasma-based genetic testing in CRC patients. The high concordance of actionable gene mutations and the increased detection of high TMB suggest that this approach may guide treatment decisions more effectively. The integration of ctDNA analysis in clinical practice may improve the precision and efficacy of treatment strategies for CRC patients.

### Supplementary Information

Below is the link to the electronic supplementary material.

Supplementary file1 (DOCX 21 kb)

Supplementary file2 (DOCX 19 kb)

Supplementary file3 (DOCX 27 kb)

Supplementary file4 (DOCX 17 kb)

## Data Availability

The datasets generated during and/or analysed during the current study are available from the corresponding author upon reasonable request.
