# Supplementary material for: Plasma ctDNA enhances the tissue-based detection of oncodriver mutations in colorectal cancer
Source: Clin Transl Oncol. 2024 May 22;26(8):1976–87. doi: 10.1007/s12094-024-03422-7 (PMC11249419; doi:10.1007/s12094-024-03422-7)
Supplement: Supplementary file 1 — Supplementary file1 (DOCX 21 kb) [file 12094_2024_3422_MOESM1_ESM.docx]

**Plasma** **ctDNA enhances the tissue-based detection of oncodriver mutations in colorectal cancer**

Wei Wang^1#^, Yisen Huang^2#^, Jianqiao Kong^3#^, Lin Lu^4^, Qianxiu Liao^5^, Jingtao Zhu^6^, Tinghao Wang^6^, Linghua Yan^7^, Min Dai^8*^, Zhan Chen^9*^, Jun You^10*^

^1^ The First People’s Hospital of Foshan, Foshan 528000, Guangdong, China

^2^ Department of Gastrointestinal Surgery, Quanzhou First Hospital Affiliated to Fujian Medical University, Quanzhou 362002, Fujian, China

^3^ Department of General Surgery, Xiangyang No.1 People's Hospital, Hubei University of Medicine, Xiangyang 441000, Hubei, China

^4^ Department of Gastrointestinal Surgery, People's Hospital of Ningxia, Yinchuan 750002, Ningxia, China

^5^ Department of Laboratory Medicine, Chengdu First People’s Hospital, Chengdu, Sichuan 610041, China

^6^The Third Clinical Medical College, Fujian Medical University, Fujian 361001, Xiamen, China.

^7^ Shanghai Tongshu Biotech Co Ltd, Shanghai 201900, China

^8^ Department of Pathology, Wuhu Hospital, East China Normal University (The Second People's Hospital, Wuhu), Wuhu 241000, Anhui, China

^9^ Department of General Surgery, Chenggong Hospital of Xiamen University School of Medicine, Fujian 361001, Xiamen, China

^10^ Department of Gastrointestinal Oncology Surgery, Cancer Center, The First Affiliated Hospital of Xiamen University, School of Medicine, Xiamen University, Fujian 361001, Xiamen, China

# Wei Wang, Yisen Huang and Jianqiao Kong contributed equally to this work.

**Correspondence**

Min Dai, Department of Pathology, Wuhu Hospital, East China Normal University (The Second People's Hospital, Wuhu), Email: Daimin0123@163.com

Zhan Chen, Department of Gastrointestinal Oncology Surgery, Cancer Center, The First Affiliated Hospital of Xiamen University, Xiamen, Fujian 361001, China. Email: [8985913@qq.com](mailto:8985913@qq.com)

Jun You, Department of Gastrointestinal Oncology Surgery, Cancer Center, The First Affiliated Hospital of Xiamen University, School of Medicine, Xiamen University, Xiamen, Fujian 361001, China. Email: youjun@xmu.edu.cn

**Running title**: ctDNA enhances the tissue-based detection in CRC

**Guarantor of the article:** Jun You

**Supplementary Table 1. Detected oncogenic mutation types.**

| **Mutated genes** | **Detected mutations** |
| --- | --- |
| **APC** | p.Arg213Ter, p.Arg216Ter, p.Asp1318fs, p.Asp1394Ter, p.Gln1291Ter, p.Glu1306fs, p.Glu1353Ter, p.Glu1379Ter, p.Glu1554fs, p.Pro1319fs, p.Ser1346Ter, p.Ser457Ter, p.Arg1450Ter, p.Arg232Ter, p.Arg805Ter, p.Arg876Ter, p.Gln1367Ter, p.Gln1447Ter, p.Leu669fs, p.Ser1356Ter, p.Arg283Ter, p.Asp1138fs, p.Gln1406fs, p.Gln1406Ter, p.Gln1429Ter, p.Glu1156fs, p.Glu1309AspfsTer4, p.Glu1494fs, p.Glu1550Ter, p.Gly1499fs, p.Trp699Ter, p.Tyr935_Asn936delinsTer, p.Tyr825Ter, p.Pro1439fs, p.Arg1114Ter, p.Arg554Ter, p.Cys1249fs, p.Gln1444Ter, p.Glu1552Ter, p.Lys1462fs, p.Ser1400Ter |
| **TP53** | p.Arg158Leu, p.Tyr220Cys, p.Arg175His, p.Arg196Ter, p.Arg213Ter, p.Arg248Gln, p.Arg248Trp, p.Arg306Ter, p.Arg342Ter, p.Glu339Ter, p.Gly245Ser, p.His168Arg, p.Ser241fs, p.Arg273His, p.Arg273Leu, p.Arg282Trp, p.Asn235fs, p.Cys176Phe, p.Cys275Trp, p.Gln165Ter, p.Gln38Ter, p.Gly105Ser, p.Gly199Ter, p.Leu145Pro, p.Leu93fs, p.Tyr205Ter, p.Ala161Thr, p.Arg273Cys, p.Glu180Lys, p.Pro301fs, p.Phe109fs, p.Arg196Gln, p.Arg273Ser |
| **KRAS** | p.Ala146Thr, p.Gln22Lys, p.Gly12Ala, p.Gly12Asp, p.Gly12Cys, p.Gly12Val, p.Gly13Asp, p.Lys117Asn, p.Gly12Arg, p.Gly12Ser, p.Gly13Ser |
| **PIK3CA** | p.Glu545Lys, p.Cys420Arg, p.Glu542Lys, p.Glu542Val, p.Glu110del, p.His1047Arg |
| **BRAF** | p.Val600Glu |
| **EGFR** | CNV amplification, p.Gly719Ala |
| **PTEN** | p.Cys211Ter, p.Leu265fs, p.Arg130Gln, p.Val317fs, p.Arg233Ter |
| **ERBB2** | p.Gly776Ser, CNV amplification, p.Arg678Gln, p.Val842Ile |
| **CTNNB1** | p.Ser45Phe, p.Thr41Ala, p.Trp383Arg |
| **MET** | CNV amplification |
| **FBXW7** | p.Arg224Ter, p.Arg465His, p.Arg465Cys, p.Arg367Ter |
| **NRAS** | p.Gln61His, p.Gly13Asp |
| **BRCA1** | p.Lys654fs, p.Ser988Ter, p.Gly948Ter, p.Thr1691Lys |
| **BRCA2** | p.Thr966fs, p.Thr967fs, p.Arg2336His |
| **FGFR1** | CNV amplification |
| **HRAS** | p.Gly12Asp, p.Gln61Lys |
| **AKT1** | p.Glu17Lys |
| **PLOE** | p.Val1446fs, p.Val411Leu |
| **NF1** | p.Arg1534Ter |
| **CDK4** | CNV amplification |
| **NBN** | p.Arg466fs |
| **FGFR3** | CNV amplification |
| **KIT** | p.Ala502Ser |
| **DNMT3A** | p.Leu566Ter, p.Ala259LeufsTer57 |
| **RB1** | p.Glu458Ter |
| **IDH1** | p.Arg132Ser |
