# Supplementary material for: Plasma ctDNA enhances the tissue-based detection of oncodriver mutations in colorectal cancer
Source: Clin Transl Oncol. 2024 May 22;26(8):1976–87. doi: 10.1007/s12094-024-03422-7 (PMC11249419; doi:10.1007/s12094-024-03422-7)
Supplement: Supplementary file 2 — Supplementary file2 (DOCX 19 kb) [file 12094_2024_3422_MOESM2_ESM.docx]

**Plasma** **ctDNA enhances the tissue-based detection of oncodriver mutations in colorectal cancer**

Wei Wang^1#^, Yisen Huang^2#^, Jianqiao Kong^3#^, Lin Lu^4^, Qianxiu Liao^5^, Jingtao Zhu^6^, Tinghao Wang^6^, Linghua Yan^7^, Min Dai^8*^, Zhan Chen^9*^, Jun You^10*^

^1^ The First People’s Hospital of Foshan, Foshan 528000, Guangdong, China

^2^ Department of Gastrointestinal Surgery, Quanzhou First Hospital Affiliated to Fujian Medical University, Quanzhou 362002, Fujian, China

^3^ Department of General Surgery, Xiangyang No.1 People's Hospital, Hubei University of Medicine, Xiangyang 441000, Hubei, China

^4^ Department of Gastrointestinal Surgery, People's Hospital of Ningxia, Yinchuan 750002, Ningxia, China

^5^ Department of Laboratory Medicine, Chengdu First People’s Hospital, Chengdu, Sichuan 610041, China

^6^The Third Clinical Medical College, Fujian Medical University, Fujian 361001, Xiamen, China.

^7^ Shanghai Tongshu Biotech Co Ltd, Shanghai 201900, China

^8^ Department of Pathology, Wuhu Hospital, East China Normal University (The Second People's Hospital, Wuhu), Wuhu 241000, Anhui, China

^9^ Department of General Surgery, Chenggong Hospital of Xiamen University School of Medicine, Fujian 361001, Xiamen, China

^10^ Department of Gastrointestinal Oncology Surgery, Cancer Center, The First Affiliated Hospital of Xiamen University, School of Medicine, Xiamen University, Fujian 361001, Xiamen, China

# Wei Wang, Yisen Huang and Jianqiao Kong contributed equally to this work.

**Correspondence**

Min Dai, Department of Pathology, Wuhu Hospital, East China Normal University (The Second People's Hospital, Wuhu), Email: Daimin0123@163.com

Zhan Chen, Department of Gastrointestinal Oncology Surgery, Cancer Center, The First Affiliated Hospital of Xiamen University, Xiamen, Fujian 361001, China. Email: [8985913@qq.com](mailto:8985913@qq.com)

Jun You, Department of Gastrointestinal Oncology Surgery, Cancer Center, The First Affiliated Hospital of Xiamen University, School of Medicine, Xiamen University, Xiamen, Fujian 361001, China. Email: youjun@xmu.edu.cn

**Running title**: ctDNA enhances the tissue-based detection in CRC

**Guarantor of the article:** Jun You

**Supplementary Table 2. Significance of tissue combined with ctDNA for positive mutation rate (N=76).**

|  |  | Tissue+ | Tissue- | Total | Positive mutation rate | | |
| --- | --- | --- | --- | --- | --- | --- | --- |
|  |  |  |  |  | Tissue | Combination | *P* value |
| *KRAS* | Combination+ | 36 | 8 | 44 | 47.37% | 57.89% | 0.008 |
|  | Combination - | 0 | 32 | 32 |  |  |  |
|  | Total | 36 | 40 | 76 |  |  |  |
| *NRAS* | Combination+ | 2 | 0 | 2 | 2.63% | 2.63% | 1.000 |
|  | Combination - | 0 | 74 | 74 |  |  |  |
|  | Total | 2 | 74 | 76 |  |  |  |
| *BRAF* | Combination+ | 6 | 1 | 7 | 7.89% | 9.21% | 1.000 |
|  | Combination - | 0 | 69 | 69 |  |  |  |
|  | Total | 6 | 70 | 76 |  |  |  |
| *PIK3CA* | Combination+ | 8 | 1 | 9 | 10.53% | 11.84% | 1.000 |
|  | Combination - | 0 | 67 | 67 |  |  |  |
|  | Total | 8 | 68 | 76 |  |  |  |
| *ERBB2* | Combination+ | 5 | 0 | 5 | 6.58% | 6.58% | 1.000 |
|  | Combination - | 0 | 71 | 71 |  |  |  |
|  | Total | 5 | 71 | 76 |  |  |  |
| Merge | Combination+ | 48 | 8 | 56 | 63.16% | 73.68% | 0.008 |
|  | Combination - | 0 | 20 | 20 |  |  |  |
|  | Total | 48 | 28 | 76 |  |  |  |
| Others | Combination+ | 69 | 1 | 70 | 90.79% | 92.11% | 1.000 |
|  | Combination - | 0 | 6 | 6 |  |  |  |
|  | Total | 69 | 7 | 76 |  |  |  |
