# Supplementary material for: Plasma ctDNA enhances the tissue-based detection of oncodriver mutations in colorectal cancer
Source: Clin Transl Oncol. 2024 May 22;26(8):1976–87. doi: 10.1007/s12094-024-03422-7 (PMC11249419; doi:10.1007/s12094-024-03422-7)
Supplement: Supplementary file 3 — Supplementary file3 (DOCX 27 kb) [file 12094_2024_3422_MOESM3_ESM.docx]

**Plasma** **ctDNA enhances the tissue-based detection of oncodriver mutations in colorectal cancer**

Wei Wang^1#^, Yisen Huang^2#^, Jianqiao Kong^3#^, Lin Lu^4^, Qianxiu Liao^5^, Jingtao Zhu^6^, Tinghao Wang^6^, Linghua Yan^7^, Min Dai^8*^, Zhan Chen^9*^, Jun You^10*^

^1^ The First People’s Hospital of Foshan, Foshan 528000, Guangdong, China

^2^ Department of Gastrointestinal Surgery, Quanzhou First Hospital Affiliated to Fujian Medical University, Quanzhou 362002, Fujian, China

^3^ Department of General Surgery, Xiangyang No.1 People's Hospital, Hubei University of Medicine, Xiangyang 441000, Hubei, China

^4^ Department of Gastrointestinal Surgery, People's Hospital of Ningxia, Yinchuan 750002, Ningxia, China

^5^ Department of Laboratory Medicine, Chengdu First People’s Hospital, Chengdu, Sichuan 610041, China

^6^The Third Clinical Medical College, Fujian Medical University, Fujian 361001, Xiamen, China.

^7^ Shanghai Tongshu Biotech Co Ltd, Shanghai 201900, China

^8^ Department of Pathology, Wuhu Hospital, East China Normal University (The Second People's Hospital, Wuhu), Wuhu 241000, Anhui, China

^9^ Department of General Surgery, Chenggong Hospital of Xiamen University School of Medicine, Fujian 361001, Xiamen, China

^10^ Department of Gastrointestinal Oncology Surgery, Cancer Center, The First Affiliated Hospital of Xiamen University, School of Medicine, Xiamen University, Fujian 361001, Xiamen, China

# Wei Wang, Yisen Huang and Jianqiao Kong contributed equally to this work.

**Correspondence**

Min Dai, Department of Pathology, Wuhu Hospital, East China Normal University (The Second People's Hospital, Wuhu), Email: Daimin0123@163.com

Zhan Chen, Department of Gastrointestinal Oncology Surgery, Cancer Center, The First Affiliated Hospital of Xiamen University, Xiamen, Fujian 361001, China. Email: [8985913@qq.com](mailto:8985913@qq.com)

Jun You, Department of Gastrointestinal Oncology Surgery, Cancer Center, The First Affiliated Hospital of Xiamen University, School of Medicine, Xiamen University, Xiamen, Fujian 361001, China. Email: youjun@xmu.edu.cn

**Running title**: ctDNA enhances the tissue-based detection in CRC

**Guarantor of the article:** Jun You

**Supplementary Table 3.** **Concordance of all detected genes between ctDNA and tumor-tissue (N=76).**

|  |  | Tissue+ | Tissue- | Total | PPV | NPV | Sensitivity | Positive mutation rate | | |
| --- | --- | --- | --- | --- | --- | --- | --- | --- | --- | --- |
|  |  |  |  |  |  |  |  | Tissue | ctDNA | Combination |
| *KRAS* | ctDNA+ | 25 | 8 | 33 | 75.76% | 74.42% | 75.00% | 47.37% | 43.42% | 57.89% |
|  | ctDNA- | 11 | 32 | 43 |  |  |  |  |  |  |
|  | Total | 36 | 40 | 76 |  |  |  |  |  |  |
| *NRAS* | ctDNA+ | 2 | 0 | 2 | 100% | 100% | 100% | 2.63% | 2.63% | 2.63% |
|  | ctDNA- | 0 | 74 | 74 |  |  |  |  |  |  |
|  | Total | 2 | 74 | 76 |  |  |  |  |  |  |
| *BRAF* | ctDNA+ | 4 | 1 | 5 | 80.00% | 97.18% | 71.43% | 7.89% | 6.58% | 9.21% |
|  | ctDNA- | 2 | 69 | 71 |  |  |  |  |  |  |
|  | Total | 6 | 70 | 76 |  |  |  |  |  |  |
| *PIK3CA* | ctDNA+ | 2 | 1 | 3 | 66.67% | 91.78% | 33.33% | 10.53% | 3.95% | 11.84% |
|  | ctDNA- | 6 | 67 | 73 |  |  |  |  |  |  |
|  | Total | 8 | 68 | 76 |  |  |  |  |  |  |
| *ERBB2* | ctDNA+ | 1 | 0 | 1 | 100% | 94.67% | 20% | 6.58% | 1.32% | 6.58% |
|  | ctDNA- | 4 | 71 | 75 |  |  |  |  |  |  |
|  | Total | 5 | 71 | 76 |  |  |  |  |  |  |
| *TP53* | ctDNA+ | 13 | 4 | 17 | 76.47% | 50.85% | 36.96% | 55.26% | 22.37% | 60.53% |
|  | ctDNA- | 29 | 30 | 59 |  |  |  |  |  |  |
|  | Total | 42 | 34 | 76 |  |  |  |  |  |  |
| *APC* | ctDNA+ | 15 | 0 | 15 | 100% | 52.46% | 34.09% | 57.89% | 19.74% | 57.89% |
|  | ctDNA- | 29 | 32 | 61 |  |  |  |  |  |  |
|  | Total | 44 | 32 | 76 |  |  |  |  |  |  |
| *BRCA1* | ctDNA+ | 0 | 2 | 2 | 0% | 97.30% | 50% | 2.63% | 2.63% | 5.26% |
|  | ctDNA- | 2 | 72 | 74 |  |  |  |  |  |  |
|  | Total | 2 | 74 | 76 |  |  |  |  |  |  |
| *BRCA2* | ctDNA+ | 1 | 0 | 1 | 100% | 98.67% | 50% | 2.63% | 1.32% | 2.63% |
|  | ctDNA- | 1 | 74 | 75 |  |  |  |  |  |  |
|  | Total | 2 | 74 | 76 |  |  |  |  |  |  |
| *CTNNB1* | ctDNA+ | 2 | 0 | 2 | 100% | 97.30% | 50% | 5.26% | 2.63% | 5.26% |
|  | ctDNA- | 2 | 72 | 74 |  |  |  |  |  |  |
|  | Total | 4 | 72 | 76 |  |  |  |  |  |  |
| *FGFR1* | ctDNA+ | 0 | 2 | 2 | 0% | 97.30% | 50% | 2.63% | 2.63% | 5.26% |
|  | ctDNA- | 2 | 72 | 74 |  |  |  |  |  |  |
|  | Total | 2 | 74 | 76 |  |  |  |  |  |  |
| *FGFR3* | ctDNA+ | 0 | 1 | 1 | 0% | 100% | 100% | 0% | 1.32% | 1.32% |
|  | ctDNA- | 0 | 75 | 75 |  |  |  |  |  |  |
|  | Total | 0 | 76 | 76 |  |  |  |  |  |  |
| *HRAS* | ctDNA+ | 0 | 1 | 1 | 0% | 98.67% | 50% | 1.32% | 1.32% | 2.63% |
|  | ctDNA- | 1 | 74 | 75 |  |  |  |  |  |  |
|  | Total | 1 | 75 | 76 |  |  |  |  |  |  |
| *PTEN* | ctDNA+ | 1 | 0 | 1 | 100% | 93.33% | 16.67% | 7.89% | 1.32% | 9.21% |
|  | ctDNA- | 5 | 70 | 75 |  |  |  |  |  |  |
|  | Total | 6 | 70 | 76 |  |  |  |  |  |  |
| *KIT* | ctDNA+ | 0 | 1 | 1 | 0% | 100% | 100% | 0% | 1.32% | 1.32% |
|  | ctDNA- | 0 | 75 | 75 |  |  |  |  |  |  |
|  | Total | 0 | 76 | 76 |  |  |  |  |  |  |
| *AKT1* | ctDNA+ | 0 | 0 | 0 | 0% | 98.68% | 0% | 1.32% | 0% | 1.32% |
|  | ctDNA- | 1 | 75 | 76 |  |  |  |  |  |  |
|  | Total | 1 | 75 | 76 |  |  |  |  |  |  |
| *DNMT3A* | ctDNA+ | 0 | 2 | 2 | 0% | 100% | 100% | 0% | 2.63% | 2.63% |
|  | ctDNA- | 0 | 74 | 74 |  |  |  |  |  |  |
|  | Total | 0 | 76 | 76 |  |  |  |  |  |  |
| *EGFR* | ctDNA+ | 0 | 3 | 3 | 0% | 97.26% | 60% | 2.63% | 3.95% | 6.58% |
|  | ctDNA- | 2 | 71 | 73 |  |  |  |  |  |  |
|  | Total | 2 | 74 | 76 |  |  |  |  |  |  |
| *NF1* | ctDNA+ | 0 | 0 | 0 | 0% | 98.68% | 0% | 1.32% | 0% | 1.32% |
|  | ctDNA- | 1 | 75 | 76 |  |  |  |  |  |  |
|  | Total | 1 | 75 | 76 |  |  |  |  |  |  |
| *POLE* | ctDNA+ | 0 | 1 | 1 | 0% | 98.67% | 50% | 1.32% | 1.32% | 2.63% |
|  | ctDNA- | 1 | 74 | 75 |  |  |  |  |  |  |
|  | Total | 1 | 75 | 76 |  |  |  |  |  |  |
| *RB1* | ctDNA+ | 0 | 1 | 1 | 0% | 100% | 100% | 0% | 1.32% | 1.32% |
|  | ctDNA- | 0 | 75 | 75 |  |  |  |  |  |  |
|  | Total | 0 | 76 | 76 |  |  |  |  |  |  |
| *CDK4* | ctDNA+ | 0 | 0 | 0 | 0% | 98.68% | 0% | 1.32% | 0% | 1.32% |
|  | ctDNA- | 1 | 75 | 76 |  |  |  |  |  |  |
|  | Total | 1 | 75 | 76 |  |  |  |  |  |  |
| *FBXW7* | ctDNA+ | 0 | 0 | 0 | 0% | 96.05% | 0% | 3.95% | 0% | 3.95% |
|  | ctDNA- | 3 | 73 | 76 |  |  |  |  |  |  |
|  | Total | 3 | 73 | 76 |  |  |  |  |  |  |
| *MET* | ctDNA+ | 0 | 0 | 0 | 0% | 94.74% | 0% | 5.26% | 0% | 5.26% |
|  | ctDNA- | 4 | 72 | 76 |  |  |  |  |  |  |
|  | Total | 4 | 72 | 76 |  |  |  |  |  |  |
| *NBN* | ctDNA+ | 0 | 0 | 0 | 0% | 98.68% | 0% | 1.32% | 0% | 1.32% |
|  | ctDNA- | 1 | 75 | 76 |  |  |  |  |  |  |
|  | Total | 1 | 75 | 76 |  |  |  |  |  |  |
| *IDH1* | ctDNA+ | 0 | 1 | 1 | 0% | 100% | 100% | 0% | 1.32% | 1.32% |
|  | ctDNA- | 0 | 75 | 75 |  |  |  |  |  |  |
|  | Total | 0 | 76 | 76 |  |  |  |  |  |  |
