# Supplementary material for: Plasma ctDNA enhances the tissue-based detection of oncodriver mutations in colorectal cancer
Source: Clin Transl Oncol. 2024 May 22;26(8):1976–87. doi: 10.1007/s12094-024-03422-7 (PMC11249419; doi:10.1007/s12094-024-03422-7)
Supplement: Supplementary file 4 — Supplementary file4 (DOCX 17 kb) [file 12094_2024_3422_MOESM4_ESM.docx]

**Plasma** **ctDNA enhances the tissue-based detection of oncodriver mutations in colorectal cancer**

Wei Wang^1#^, Yisen Huang^2#^, Jianqiao Kong^3#^, Lin Lu^4^, Qianxiu Liao^5^, Jingtao Zhu^6^, Tinghao Wang^6^, Linghua Yan^7^, Min Dai^8*^, Zhan Chen^9*^, Jun You^10*^

^1^ The First People’s Hospital of Foshan, Foshan 528000, Guangdong, China

^2^ Department of Gastrointestinal Surgery, Quanzhou First Hospital Affiliated to Fujian Medical University, Quanzhou 362002, Fujian, China

^3^ Department of General Surgery, Xiangyang No.1 People's Hospital, Hubei University of Medicine, Xiangyang 441000, Hubei, China

^4^ Department of Gastrointestinal Surgery, People's Hospital of Ningxia, Yinchuan 750002, Ningxia, China

^5^ Department of Laboratory Medicine, Chengdu First People’s Hospital, Chengdu, Sichuan 610041, China

^6^The Third Clinical Medical College, Fujian Medical University, Fujian 361001, Xiamen, China.

^7^ Shanghai Tongshu Biotech Co Ltd, Shanghai 201900, China

^8^ Department of Pathology, Wuhu Hospital, East China Normal University (The Second People's Hospital, Wuhu), Wuhu 241000, Anhui, China

^9^ Department of General Surgery, Chenggong Hospital of Xiamen University School of Medicine, Fujian 361001, Xiamen, China

^10^ Department of Gastrointestinal Oncology Surgery, Cancer Center, The First Affiliated Hospital of Xiamen University, School of Medicine, Xiamen University, Fujian 361001, Xiamen, China

# Wei Wang, Yisen Huang and Jianqiao Kong contributed equally to this work.

**Correspondence**

Min Dai, Department of Pathology, Wuhu Hospital, East China Normal University (The Second People's Hospital, Wuhu), Email: Daimin0123@163.com

Zhan Chen, Department of Gastrointestinal Oncology Surgery, Cancer Center, The First Affiliated Hospital of Xiamen University, Xiamen, Fujian 361001, China. Email: [8985913@qq.com](mailto:8985913@qq.com)

Jun You, Department of Gastrointestinal Oncology Surgery, Cancer Center, The First Affiliated Hospital of Xiamen University, School of Medicine, Xiamen University, Xiamen, Fujian 361001, China. Email: youjun@xmu.edu.cn

**Running title**: ctDNA enhances the tissue-based detection in CRC

**Guarantor of the article:** Jun You

**Table S4. Concordance of TMB status** **between ctDNA and tumor-tissue (N=64).**

|  |  | Tissue | | | NPV | Specificity | Positive detection rate | | |
| --- | --- | --- | --- | --- | --- | --- | --- | --- | --- |
|  |  | TMB-H | TMB-L | Total |  |  | Tissue | ctDNA | Combination |
| ctDNA  (N=64) | TMB-H | 0 | 5 | 5 | 89.58% | 72.88% | 25% | 7.81% | **32.81%** |
|  | TMB-L | 16 | 43 | 59 |  |  |  |  |  |
|  | Total | 16 | 48 | 64 |  |  |  |  |  |
